# Inequalities in time to cancer treatment initiation over a decade in Brazil

**DOI:** 10.11606/s1518-8787.2026060006943

**Published:** 2026-03-16

**Authors:** Matheus de Abreu, Maria Emília Mota, Dandara Menezes de Araujo Oliveira, Yuri de Lima Medeiros, Maria Stella Moreira, Fábio Abreu Alves, Maria Paula Curado

**Affiliations:** I A.C. Camargo Cancer Center. Centro Internacional de Pesquisa. São Paulo, SP, Brasil; II Universidade de São Paulo. Faculdade de Odontologia. Departamento de Estomatologia. São Paulo, SP, Brasil; III A.C. Camargo Cancer Center. Departamento de Estomatologia. São Paulo, SP, Brasil

**Keywords:** Neoplasms, Uterine Cervical Neoplasms, Colorectal Neoplasms, Thyroid Neoplasms, Central Nervous System Neoplasms, Treatment Delay, Sociodemographic Factors, Health Inequities, Health Status Disparities

## Abstract

**OBJECTIVE:**

To evaluate access to treatment for cervical (CVC), colorectal (CRC), thyroid (CT), and central nervous system (CCNS) cancers that differ in incidence, prognosis, early detection program, and in their requirements for high-complexity and technologically advanced treatments

**METHODS:**

Data from Integrador RHC INCA (2013–2022) were extracted. Sociodemographic and clinic characteristics and time diagnosis-to-treatment were analyzed using multinomial logistic regression and cumulative incidence function

**RESULTS:**

395,225 cases of cancers were identified, CRC = 168,951, CVC = 141,189, CT = 57,755 and CCNS = 27,330. Patients with CCNS and CRC had higher cumulative probability (CPob) of treatment initiation within 60 days compared to CVC/CT. The North region was less likely to receive treatment within 30 days for all cancers (odds ratio — OR: CCNS = 0.34; CVC = 0.30; CT = 0.17; CRC = 0.49). Radiotherapy showed a lower chance of earlier treatment for all cancers (94% lower CCNS; CVC = 48%; CRC = 81%). There was a greater chance of treatment initiation within 30 days for patients with higher education for CVC (OR = 1.42); CT (OR = 2.09). White individuals demonstrated a consistently higher CPob of treatment initiation at 60 days for all cancers compared to Black (CCNS: 60% *versus* 49%; CVC: 37% *versus* 29%; CT: 32% *versus* 21%; CRC: 52% *versus* 45%). For CT, males had a higher CPob of treatment 37% in males compared to 30% in females at 60 days). For CRC, CPob of treatment initiation at 60 days was higher among individuals at younger (60% compared to 46% for those aged > 69 years) and clinical stage IV (56% compared to stage 1 — 45%).

**CONCLUSION:**

In Brazil, patients requiring multimodal treatments or therapies demanding technology, such as radiotherapy or chemotherapy, faced longer delays. Regional and sociodemographic disparities persist, with timely access to cancer treatment limited in socioeconomically disadvantaged regions, such as the North and Northeast, among Black patients and individuals with lower education.

## INTRODUCTION

 Brazil is a country with social, economic and health access inequalities^1^. The country has a population of over 205 million and is an upper-middle income country according to the World Bank Classification^2^. These regional inequalities can affect the health of the population by limiting access to and utilization of health services^3^. The main determinants of health inequity are socioeconomic status, social class, sex, race/ethnicity and geographic location^4^. 

 The Brazilian National Health System was implemented in Brazil in 1988 and constitutes a complex dynamic public health system grounded on the principle of health as a right of citizens and duty of the state^5^. In 2012, Law 12,732/2012 was enacted, stipulating a maximum permitted time of 60 days, nationwide, from cancer diagnosis to treatment initiation^6^. Data from the Brazilian National Cancer Institute (INCA) and the System of Hospital Cancer Registries show this goal is often not achieved, with major regional disparities in average time in days to treatment initiation^7^. This delay in access can negatively impact treatment outcomes and patient prognosis. Factors such as low educational level, financial difficulties, and geographic distance to cancer treatment centers are cited as significant barriers^8^. 

 There were an estimated 704 thousand new cases of cancer in Brazil during the 2023–2025 triennium. Cervical cancer (CVC) is the second most incident carcinoma in the North and Northeast, with an estimated 17 thousand new cases annually in the 2023–2025 triennium. Colorectal cancer (CRC) is the second most incident neoplasm in men, with higher rates in the Southeast region of Brazil. Estimates suggest around 45,630 cases for each year of the 2023–2025 triennium, corresponding to an estimated risk of 20.78 new cases per 100 thousand men and 21.41 per 100 thousand women^9^. Cancer of the central nervous system (CCNS) requires high-complexity services for diagnoses and treatment^10^, ranking as the 8^th^ most common cancer in the Mid-West and North regions, with an estimated 11,490 cases in 2023–2025 nationally, comprising 6,110 cases in men and 5,380 in women. Cancer of the thyroid (CT) is the third-most-common type of cancer among women in the Southeast and Northeast regions, with an estimated 16,660 new cases for each year of the triennium in Brazil^9^. 

 Early diagnosis and treatment of cancer can often allow a unimodal approach to be used^11^. Timely treatment is more effective, has fewer long-term secondary effects, and is lower cost for health systems^12^. Disparities in time between diagnosis and treatment of cancer in Brazil correspond to major regional and socioeconomic inequalities. Monitoring these health disparities is crucial for tracking treatment on a regional level^1^. 

 The literature addressing the interval between diagnosis and treatment initiation for CVC, CRC, CT and CCNS remains limited. Most available evidence originates from high-income countries, where healthcare systems and service delivery structures differ substantially from the Brazilian context^ 13-17^. In Brazil, existing studies are largely based on data from individual hospital centers or a single Brazilian state^18-20^, which limits the generalizability of their findings to the broader population. Moreover, the few nationwide analyses available predominantly reflect periods prior to the enactment of the law^6^ that established a regulatory framework for timely cancer treatment within the public healthcare system^8^. Consequently, a significant gap remains in the comprehensive and up-to-date evaluation of inequalities in access to timely cancer care in Brazil. 

 Considering the negative impact of treatment delays on patient outcomes, this study aimed to assess the time between diagnosis and treatment initiation in Brazil, investigating whether sociodemographic and regional disparities persist, as well as differences among therapeutic modalities with varying levels of technological complexity and financial requirements. The study compares CVC, which is included in institutionalized early detection and screening programs in Brazil^21^; CRC, which has shown a rising incidence in recent years^22^ and is difficult to treat, with a poor prognosis in advanced stages^23^; CT, which has exhibited an upward trend in incidence alongside a decline in mortality^24^; and CCNS, which requires high-complexity treatment and advanced technological resources for management in advanced stages^25^. 

## METHODS

### IntegradorRHC

 An ecological, cross-sectional study was conducted using data extracted from IntegradorRHC, a public web-based system that compiles patient information from Cancer Hospital Registries (RHCs). These registries submit their information to the national Cancer Hospital Registry database, which is managed by INCA. Through IntegradorRHC, the data are organized according to the year of the patient’s first consultation at the reporting hospital. On IntegradorRHC, cancer cases are defined based on the International Classification of Diseases for Oncology, 3^rd^ Edition (ICD-O-3). The data dictionary for IntegradorRHC is available from:  https://irhc.inca.gov.br/RHCNet/visualizaTabNetExterno.action under the Download section by selecting Enviar > Dicionário de dados. 

### Data Access and Sample Processing

 The completed data were extracted year by year from the IntegradorRHC website^26^ in .dbf file format. Data integration and processing used R programming language. An automated approach was performed employing the foreign and dplyr packages to read and merge multiple .dbf files, minimizing manual errors and ensuring efficient data integration. 

 Filters were applied during data processing. Analytical cases of patients diagnosed with CVC (C.53), CRC (C.18, C.19, and C.20), CCNS (C.70, C.71, and C.72), and CT (C.73) from 2013 to 2022 were selected. Patients with an age at diagnosis over a hundred years were excluded. 

 The variables analyzed in this study were: sex (male or female), age at diagnosis (years), ethnicity (White, Black, Brown, Asian, or Indigenous), education (years), marital status (married, separated, widowed, or single), region of residence (South, Southeast, Midwest, Northeast, or North), smoking status (smoker, former smoker, or non-smoker), alcohol consumption (drinker, former drinker, or non-drinker), cancer clinical stage (I, II, III, or IV), treatment (surgery, surgery plus other modalities, chemotherapy, radiotherapy, chemotherapy plus radiotherapy, other treatments, or no treatment), disease status after treatment (disease progression, therapeutic support, death, complete remission, partial remission, or stable disease), and the time between diagnosis (DTDIAGNO — a variable present in the dataset and described in the IntegradorRHC data dictionary) and treatment initiation (DATAINITRT variable), measured in days. Age was categorized into three groups (18–49, 50–69, and > 70 years), as were education (< 10, 10–12, and > 12 years) and time between diagnosis and treatment (0–30, 31–60, and > 60 days). All variables were checked for data structure and consistency, and cleaned or corrected as needed to ensure data quality using the dplyr and tidyr packages, along with base R functions. Clinical staging analysis was not performed for CCNS tumors due to their fundamentally different classification system compared to the TNM staging used for solid tumors. For CT, the standard treatment consists of surgery and radioiodine therapy^27^, due to missing more accurate data on radioiodine therapy; only surgical treatment was considered for CT case. Records with inconsistencies regarding topography, morphology, and age were excluded. Implausible and inconsistent values were treated as missing data. The missing data proportions are presented in Supplementary File 1^ a ^— Table 1. 

### Statistical Analysis

 A descriptive analysis of absolute and relative frequencies was performed for the entire dataset and for each cancer group, stratified by the time between diagnosis and treatment initiation. Simple and multiple multinomial logistic regression analyses were performed to calculate odds ratios (OR) with 95% confidence intervals (95%CI) using nnet package. Separate regression models were created for each cancer group. In all models, the reference category was the group with the longest time between diagnosis and treatment, defined as over 60 days. The multiple regression models included variables with a p-value < 0.20 in the simple analyses. Variable selection considered both theoretical and statistical criteria. Model selection was performed in a stepwise fashion, with a variance analysis (ANOVA) test applied after each new variable was added. The final model was determined based on these criteria:

- No change in OR > 10%;
- Improved precision at the 95%CI;
- Total degrees of freedom allowed for the outcome.

The model fit of the final model was assessed using the Hosmer-Lemeshow test from the ResourceSelection package. The code used was provided in Supplementary File 2. 

 Simple mean days elapsed between diagnosis and treatment initiation was calculated for each municipality and for Brazilian states, stratified by cancer subtype and treatment modality. The means were visualized in graphs of Brazil map using the R packages geobr, viridis, sf, cowplot, ggplot2, and rmappsher. A color scale with green indicating lower mean time between diagnosis and treatment, and red showing higher means was used. White corresponded to the neutral point of 60 days used to represent the maximum period of 60 days permitted for treatment of cancer after diagnosis, as stipulated by Law no. 12.732/2012^6^. 

 A cumulative incidence analysis was conducted to estimate the cumulative probability of treatment initiation after diagnosis, stratified by sociodemographic and clinical variables for the entire dataset and each cancer subsite, using the cuminc function from the cmprsk package. The status variable indicated the occurrence of the event (treatment initiation). To limit the influence of late observations, ensure data quality and minimize the impact of potential inconsistencies, the analysis was truncated at 360 days after diagnosis. The curves were plotted using ggplot2 and arranged with gridExtra. 

## RESULTS

**Figure 1:**
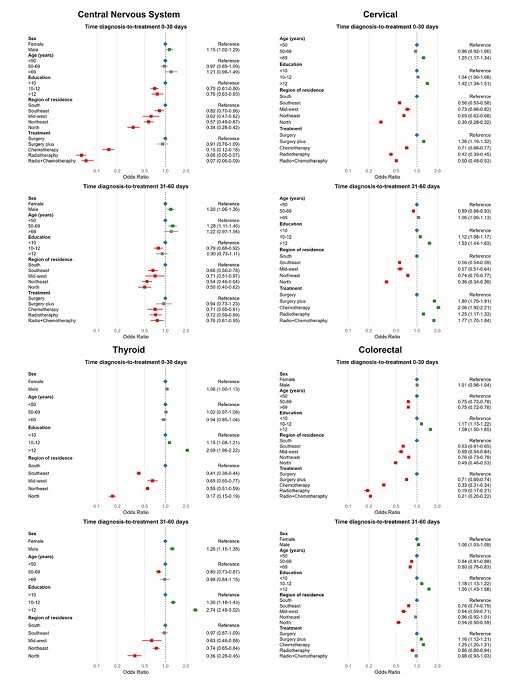
Factors associated with delays in cancer treatment initiation among patients diagnosed with cervical, colorectal, central nervous system, and thyroid cancers. IntegradorRHC — INCA, Brazil (2013–2022).

 Over a 10-year period spanning from 2013 to 2022, there were 395,225 cases of CVC, CRC, CT and CCNS in IntegradorRHC INCA. Colorectal cancer was the most frequent type, with 168,951 (42.7%) cases registered, followed by CVC (n = 141,189 — 35.7%), CT (n = 57,755 — 14.6%) and CCNS (n = 27,330 — 7.0%) (Supplementary File 1 — Table 2). Patients with CCNS and CRC had a higher cumulative probability of treatment initiation within 60 days (60 and 48%, respectively) compared to CVC and CT — approximately 31% (Supplementary File 1 — Table 7 and Figure 1). 

### Central Nervous System Cancer

 CCNS had a higher frequence in men (55.5% [n = 15,158]), and (52.3% [n = 14,285]) of patients were < 50 years. Surgery treatment was given in 26.8% (n = 7,287) of cases, radiotherapy in 18.3% (n = 4,972]) (Supplementary File 1 — Table 2). Approximately 53.1% (n = 4,338) of older patients (> 69 years) received treatment within 30 days and 28.3% (n = 1,173) at > 60 days. Around 46.2% (n = 2,914) of White patients were treated within 30 days, and only 40.2% (n = 239) of Black patients within this interval. Almost half the cases (43.3% (n = 2,111)) in the Northeast were treated at > 60 days, compared with 22.7% (n = 2,367) of cases in the Southeast region. Surgery alone and surgery plus another treatment modality were offered within 30 days in approximately 79.5% (n = 8,333) of CCNS cases, radiotherapy in 19.3% (n = 918) and chemotherapy in 35.8% (n = 737) (Table 1). 

**Table 1:** Sociodemographic, clinical characteristics and habits of patients with diagnosed of cervical, colorectal, central nervous system, and thyroid cancer. IntegradorRHC — INCA, Brazil (2013–2022).

		CCNS – n (%)			CVC – n (%)			CT – n (%)			CRC – n (%)		
		Time (diagnosis-to-treatment) in days											
		0–30	31–60	> 60	0–30	31–60	> 60	0–30	31–60	> 60	0–30	31–60	> 60
Sex													
	Male	6,757 (53.8)	2,124 (16.9)	3,667 (29.2)	-	-	-	40,43 (46.3)	1,157 (13.2)	3,538 (40.5)	23,567 (30.8)	18,295 (23.9)	34,558 (45.2)
	Female	5,342 (53.6)	1,640 (16.4)	2,993 (30.0)	2,3929 (18.7)	23,342 (18.3)	80,387 (63.0)	17,719 (45.3)	4,250 (10.9)	17,116 (43.8)	23,740 (31.6)	17,160 (22.8)	34,231 (45.6)
Age (years)													
	< 50	6,437 (53.2)	1,858 (15.4)	3,809 (31.5)	15,764 (20)	14,071 (17.9)	48,848 (62.1)	12,781 (45.4)	3,541 (12.6)	11,813 (42.0)	9,007 (33.9)	6,763 (25.4)	10,832 (40.7)
	50–69	1,324 (58.9)	388 (17.3)	534 (23.8)	1,867 (17.6)	2,146 (20.3)	6,578 (62.1)	1,307 (43.4)	292 (9.7)	1,414 (46.9)	13,586 (31.6)	9,299 (21.6)	20,163 (46.8)
	> 69	4,338 (53.1)	1,518 (18.6)	2,317 (28.3)	6,298 (16.4)	7,125 (18.6)	24,961 (65.0)	7,674 (46.0)	1,574 (9.4)	7,427 (44.5)	24,714 (30.2)	19,393 (23.7)	37,794 (46.1)
Ethnicity													
	White	2,914 (46.2)	1,356 (21.5)	2,041 (32.3)	6,397 (20.7)	6,658 (21.5)	17,888 (57.8)	5,347 (51.2)	1,050 (10.0)	4,056 (38.8)	16,386 (32.2)	12,826 (25.2)	21,753 (42.7)
	Black	239 (40.2)	112 (18.8)	244 (41.0)	938 (16.1)	1,030 (17.6)	3,875 (66.3)	513 (44.8)	63 (5.5)	568 (49.7)	1,392 (28.7)	1,088 (22.4)	2,378 (49)
	Brown	2,957 (44.1)	1,178 (17.6)	2,571 (38.3)	9,400 (16.5)	10,228 (18)	37,323 (65.5)	6,776 (41.2)	1,503 (9.1)	8,168 (49.7)	11,663 (28.9)	9,915 (24.6)	18,774 (46.5)
	Asian	30 (27.3)	14 (12.7)	66 (60.0)	202 (21.3)	172 (18.2)	573 (60.5)	143 (47.8)	26 (8.7)	130 (43.5)	250 (28.8)	216 (24.9)	401 (46.3)
	Indigenous	15 (44.1)	6 (17.6)	13 (38.2)	69 (21.6)	42 (13.2)	208 (65.2)	19 (48.7)	3 (7.7)	17 (43.6)	42 (35.0)	21 (17.5)	57 (47.5)
Education (years)													
	0–9	6,545 (55.0)	1,994 (16.8)	3,361 (28.2)	11,583 (17.3)	12,132 (18.1)	43,252 (64.6)	7,283 (44.0)	1,329 (8.0)	7,945 (48.0)	24,380 (29.8)	18,803 (23.0)	38,573 (47.2)
	10–12	1,917 (49.4)	648 (16.7)	1,314 (33.9)	5,050 (18.9)	4,881 (18.3)	16,731 (62.8)	4,884 (44.8)	1,072 (9.8)	4,950 (45.4)	8,201 (32.2)	6,358 (25.0)	10,893 (42.8)
	> 12	869 (52.4)	280 (16.9)	508 (30.7)	1,976 (23.6)	1,789 (21.3)	4,615 (55.1)	4,792 (53.3)	1,353 (15)	2,852 (31.7)	4,252 (37.4)	2,990 (26.3)	4,138 (36.4)
Marriage status													
	Married	2,538 (41.3)	1,277 (20.8)	2,326 (37.9)	7,081 (18.4)	7,529 (19.6)	23,879 (62.0)	6,443 (43.9)	1,442 (9.8)	6,805 (46.3)	15,382 (30.2)	13,265 (26.1)	22,268 (43.7)
	Separated/Viuvo	601 (45.2)	267 (20.1)	461 (34.7)	2,333 (16.6)	2,790 (19.9)	8,907 (63.5)	1,379 (43.0)	316 (9.9)	1,513 (47.2)	5,202 (29.2)	4,239 (23.8)	8,399 (47.1)
	Single	2,907 (46.9)	1,094 (17.7)	2,197 (35.4)	6,685 (16.9)	7,277 (18.4)	25,677 (64.8)	4,138 (43.1)	798 (8.3)	4,666 (48.6)	5,445 (28.2)	4,649 (24.1)	9,220 (47.7)
Region of residence													
	Mid-west	711 (56.7)	208 (16.6)	335 (26.7)	1,520 (23.5)	1,074 (16.6)	3,869 (59.9)	571 (43.6)	126 (9.6)	612 (46.8)	2,009 (29.0)	1,483 (21.4)	3,432 (49.6)
	Northeast	1,815 (37.2)	950 (19.5)	2,111 (43.3)	6,792 (17.1)	8,190 (20.6)	24,702 (62.2)	7,314 (42.5)	1,586 (9.2)	8,290 (48.2)	7,228 (28.2)	6,726 (26.3)	11,635 (45.5)
	Norht	638 (43.8)	258 (17.7)	559 (38.4)	1,879 (12.4)	2,058 (13.5)	11,260 (74.1)	501 (27.1)	168 (9.1)	1,180 (63.8)	1,482 (26.0)	1,162 (20.4)	3,047 (53.5)
	Southeast	6,702 (64.4)	1,336 (12.8)	2,367 (22.7)	8,627 (19)	7,323 (16.1)	29,437 (64.9)	9,225 (44.4)	2,975 (14.3)	8,598 (41.3)	24,010 (31.0)	17,268 (22.3)	36,254 (46.8)
	South	2,127 (48.9)	986 (22.7)	1,240 (28.5)	4,972 (24.5)	4,564 (22.5)	10,762 (53.0)	4,034 (62.7)	531 (8.3)	1,866 (29.0)	12,253 (35.0)	8,634 (24.7)	14,090 (40.3)
Smoking													
	Smoker	402 (46.9)	160 (18.7)	295 (34.4)	2,047 (16.2)	2,438 (19.3)	8,124 (64.4)	523 (42.4)	102 (8.3)	608 (49.3)	2,914 (29.9)	2,425 (24.9)	4,396 (45.2)
	Former smoker	614 (39.4)	314 (20.2)	629 (40.4)	1,675 (13.8)	2,435 (20.0)	8,042 (66.2)	753 (34.1)	244 (11.1)	1,208 (54.8)	5,157 (26.7)	4,702 (24.4)	9,443 (48.9)
	Never smoker	2,308 (40.0)	1,117 (19.3)	2,351 (40.7)	7,501 (16.2)	8,922 (19.3)	29,800 (64.5)	5,995 (43.6)	1,166 (8.5)	6,589 (47.9)	12,628 (29.5)	11,083 (25.9)	19,156 (44.7)
Drinking													
	Drinker	522 (41.0)	246 (19.3)	505 (39.7)	1,480 (13.7)	2,018 (18.6)	7,341 (67.7)	885 (37.4)	203 (8.6)	1,276 (54.0)	3,768 (28.3)	3,436 (25.8)	6,105 (45.9)
	Former drinker	385 (35.4)	195 (17.9)	507 (46.6)	974 (13.5)	1,452 (20.1)	4,786 (66.4)	341 (29.5)	133 (11.5)	680 (58.9)	2,719 (25.0)	2,708 (24.9)	5,428 (50)
	Never drinker	2,232 (40.8)	1,080 (19.7)	2,160 (39.5)	7,460 (16.3)	8,638 (18.9)	29,618 (64.8)	5,644 (44.5)	1,106 (8.7)	5,944 (46.8)	12789 (29.6)	11,049 (25.6)	19,366 (44.8)
Clinical stage													
	I	-	-	-	2,385 (17)	2,276 (16.2)	9,366 (66.8)	10,834 (50.4)	2,800 (13.0)	7,875 (36.6)	3,500 (35.5)	1,989 (20.2)	4,364 (44.3)
	II	-	-	-	2,557 (11.8)	4,581 (21.2)	14,479 (67.0)	1,398 (50.2)	177 (6.4)	1,208 (43.4)	8,584 (30.6)	6,334 (22.6)	13,108 (46.8)
	III	-	-	-	3,890 (15.3)	5,781 (22.7)	15,837 (62.1)	1,909 (45.5)	444 (10.6)	1,846 (44.0)	10,515 (27.5)	9,397 (24.6)	18,259 (47.8)
	IV	-	-	-	1,687 (19.0)	2,203 (24.8)	4,978 (56.1)	828 (39.7)	273 (13.1)	986 (47.2)	10,794 (34.0)	8,127 (25.6)	12,807 (40.4)
Treatment													
	Surgery	4,663 (79.9)	490 (8.4)	682 (11.7)	12,226 (24.3)	6,609 (13.1)	31,426 (62.5)	14,971 (49.6)	3,095 (10.2)	12,130 (40.2)	17,711 (45.1)	6,877 (17.5)	14,675 (37.4)
	Surgery plus	4,673 (79.3)	492 (8.3)	729 (12.4)	3,205 (24.8)	2,587 (20.0)	7,138 (55.2)	-	-	-	18,259 (34.7)	12,001 (22.8)	22,315 (42.4)
	Chemotherapy	737 (35.8)	436 (21.2)	886 (43.0)	1,308 (15.7)	2,014 (24.2)	5,004 (60.1)	-	-	-	6,578 (19.5)	10,041 (29.8)	17,120 (50.7)
	Radiotherapy	918 (19.3)	1,273 (26.8)	2,559 (53.9)	1,426 (11)	2,332 (17.9)	9,259 (71.1)	-	-	-	754 (13.7)	1,364 (24.8)	3,377 (61.5)
	Radio plus Chemotherapy	661 (21.8)	893 (29.4)	1,484 (48.8)	4,280 (11.4)	8,581 (22.9)	24,565 (65.6)	-	-	-	2,055 (13.9)	3,958 (26.7)	8,800 (59.4)
	Others modalities	356 (47.0)	143 (18.9)	259 (34.2)	1,102 (24.9)	1,011 (22.8)	2,314 (52.3)	-	-	-	1,499 (36.0)	980 (23.5)	1,687 (40.5)
	No treatment	66 (52.0)	24 (18.9)	37 (29.1)	216 (31.9)	117 (17.3)	344 (50.8)	-	-	-	235 (34.6)	148 (21.8)	296 (43.6)

CCNS: cancer of the central nervous system; CVC: cervical cancer; CT: cancer of the thyroid; CRC: colorectal cancer.

 On the multiple regression model, males had a greater chance of receiving treatment within 60 days (OR = 1.15 for [95%CI 0–30]; OR = 1.20 for [95%CI 31–60]). Individuals in the Northeast (OR = 0.57 [95%CI 0.49–0.67]) and North (OR = 0.34 [95%CI 0.28–0.42]) were less likely to receive treatment within 30 days compared to the South region. Cases for which radiotherapy was performed had a 94% lower chance of treatment initiation within 30 days (OR = 0.06 [95%CI 0.05–0.07]) compared to surgery (Figure 1; Supplementary File 1 — Table 4). 

**Figure 2:**
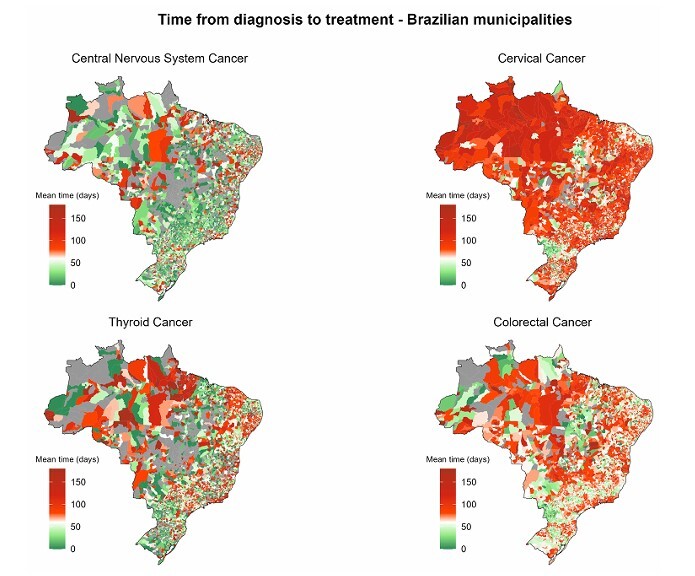
Mean time in days between diagnosis and treatment of cases of cervical, colorectal, central nervous system, and thyroid cancer by state in Brazil. IntegradorRHC — INCA, Brazil (2013–2022).

**Figure 3:**
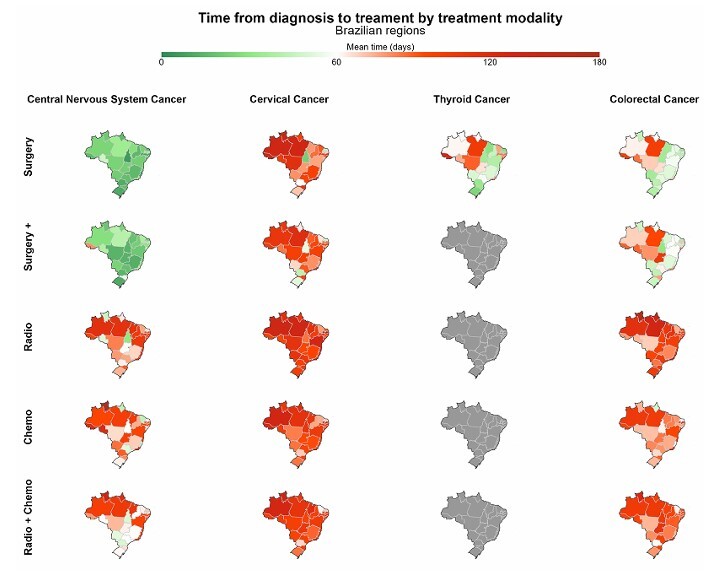
Mean time in days between diagnosis and treatment of cases of cervical, colorectal, central nervous system, and thyroid cancer by treatment modality and state in Brazil. IntegradorRHC — INCA, Brazil (2013–2022).

 Time to treatment initiation for CCNS in Brazil averaged 49 days. The shortest means werefound in Minas Gerais [30 days] and São Paulo [32 days], while the longest were in Acre[97 days], Pará, and Paraíba [83 days] (Figure 2; Supplementary File 1 — Table 5). Patientswho underwent surgery had the shortest intervals, with only three states showing averageslonger than one month (Pará and Paraíba [42 days]; Rondônia [46 days]). Radiotherapy and chemotherapy had longer intervals (radiotherapy > 100 days: Amazonas, Maranhão, Pará, and Pernambuco; chemotherapy > 100 days: Acre, Rio de Janeiro, Rondônia, and Roraima;multimodal radiotherapy plus chemotherapy > 100 days: Amapá and Roraima) (Figure 3; Supplementary File 1 — Table 6). 

**Figure 4:**
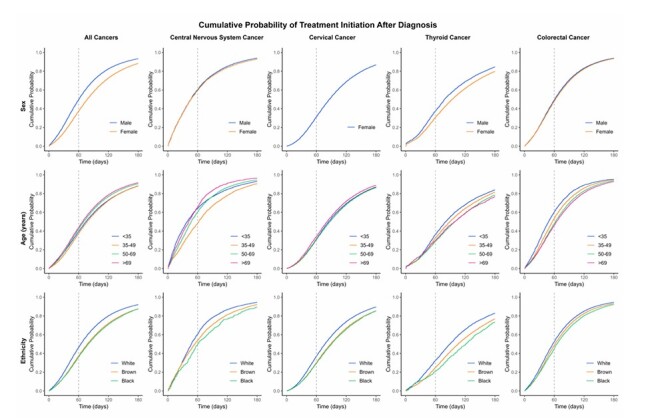
Probability of treatment initiation among patients diagnosed with cervical, colorectal, central nervous system, and thyroid cancers by sociodemographic characteristics (Cumulative Incidence Function). IntegradorRHC — INCA, Brazil (2013–2022).

**Figure 5:**
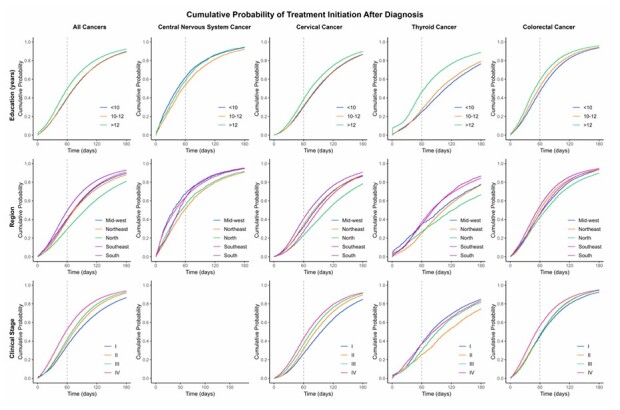
Probability of treatment initiation among patients diagnosed with cervical, colorectal, central nervous system, and thyroid cancers by sociodemographic, clinical characteristics (Cumulative Incidence Function). IntegradorRHC — INCA, Brazil (2013–2022).

 The cumulative probability of treatment initiation at 30 days was higher among individuals at the age extremes (< 35 years — 41%; > 69 years – 38%). White patients had a 60% probability at 60 days, while Black patients had only 49%. Advanced stages (III/IV) showed higher cumulative probabilities (65% and 50%, respectively) compared to early stages (I – 26%; II – 35%) (Figures 4 and 5 ; Supplementary File 1 — Table 7). 

### Cervix Uteri Cancer

 Females with CVC were 18–49 years (61.8% [n = 87,230]), Brown (59.6% [n = 63,134]), single (43.2% [n = 44,334]) and had lower educational level (65.5% [n = 73,146]). The Southeast (34.6% [n = 48,571]) and Northeast regions (32.0% [n = 44,888]) had the most CVC cases. Surgery was the most common treatment (40.0% [n = 56,020]). Treatment occurred > 60 days in 63.0% of cases [n = 80,387] (Supplementary File 1 — Table 2). In the North region, 74.1% [n = 11,260] of CVC cases were treated > 60 days, higher than in any other region of the country (South: 53.0%) (Table 1). 

 There was a greater chance of treatment initiation within 30 days for patients with higher education level (OR = 1.42 [95%CI 1.34–1.51]). Compared to the South region, all other Brazilian regions showed a lower chance of treatment initiation within 60 days, with the North region exhibiting the worst disparity (OR = 0.30 [95%CI 0.28–0.32]). Patients who underwent radiotheraphy (58%) or radiotheraphy plus chemotherapy (50%) were less likely to receive earlier treatment (< 31 days) (Figure 1; Supplementary File 1 — Table 4). 

 Time to treatment initiation for CVC in Brazil averaged 96 days. Only two states had intervals < 60 days (Rio Grande do Norte and Tocantins [59 days]) (Figure 2; Supplementary File 1 — Table 5). Regarding treatment modality, all states had averages above 60 days, except forTocantins (all modalities except radiotherapy plus chemotherapy), Rio Grande do Norte(all modalities except radiotherapy plus chemotherapy), Paraná (surgery and surgery plus),and the Federal District (surgery). The states with the longest times for chemotherapy wereAmazonas [122 days], Acre [115 days] and Amapá [109 days]; for radiotherapy: Amazonas ePará [125 days] and Sergipe [121 days]; and for multimodal radiotherapy plus chemotherapy:Roraima [138 days], Amapá [118 days] and Rio de Janeiro [114 days] (Figure 3; Supplementary File 1 — Table 6). 

 White females demonstrated a higher cumulative probability of treatment initiation at 60 days (37%) compared to Black females (29%). Higher education levels were associated with an increased probability of treatment at all time points. Patients living in the South showed a higher probability (40% at 60 days) compared to other regions (North — 23%). Advanced stages (III/IV) had higher cumulative probabilities at 60 days (36 and 42%, respectively) compared to early stages (I — 26%; II — 31%) (Figures 4 and 5; Supplementary File 1 — Table 7). 

### Thyroid Cancer

 Overall, 82.0% [n = 47,382] of CT cases occurred in women, 18–49 years (58.2% [n = 33,596]). Southeast (39.3% [n = 22,661]) and Northeast (39.2% [n = 22,608]) had most of the cases. Clinical stage I was the most frequent (69.8% [n = 24,084]) (Supplementary File 1 — Table 2). Time-to-treatment was > 60 days in 49.7% in Black and Browns patients and 38.8% in Whites. Treatment was received within 30 days in 62.7% [n = 4,034] of patients in the South and only in 27.1% [n = 501] in the North region (Table 1). 

 Patients with higher educational levels had twice the probability of receiving treatment within 30 days. Compared to the South, all other Brazilian regions had a lower chance of earlier treatment, the North being worst scenario (OR = 0.17 [95%CI 0.15–0.19]) followed by the Southeast (OR = 0.41 [95%CI 0.38–0.44]) (Figure 1; Supplementary File 1 — Table 4). 

 Time to treatment initiation for CT in Brazil averaged 69 days. Eight states had means <60 days (Tocantins and Rio Grande do Sul [33 days]; Maranhão [40 days] Paraná and RioGrande do Norte [42 days], Paraíba [43 days], Santa Catarina [47 days] and Minas Gerais[55 days]. The longest intervals were observed in Acre [138 days], Sergipe [123 days] andAmapá [118 days]) (Figure 2; Supplementary File 1 — Table 5). 

 Males had a higher cumulative probability of treatment at all time points (37% in males compared to 30% in females at 60 days), as did White individuals (32% in White individuals compared to 21% in Black individuals at 60 days). The cumulative probability of treatment at 60 days was 46% among those with more than 12 years of education, compared to only 25% among those with less than ten years. The Southeast region had the highest probability at 60 days (37%) compared to only 25% in the Northeast region (Figures 4 and 5; Supplementary File 1 — Table 7). 

### Colorectal Cancer

 Regarding CRC patients, 53.5% (n = 90,309) of cases were aged > 69 years, whereas, geographically, 50.8% (n = 85,298) resided in the Southeast region. Advanced stage (III and IV) was 64.8% of CRC cases (n = 75,090). Surgery plus was the elected treatment in 33.2% (n = 55,673) (Supplementary File 1 — Table 2). By region, more than a half of cases (53.5% [n = 3,047]) were treated at > 60 days in the North, followed by the Mid-West region (49.6% [n = 3,432]). Around 45.0% (n = 17,711) of surgical cases received treatment within 30 days, compared with 13.7% of cases indicated for radiotherapy. More than half of the CRC patients (59.4% [n = 40,788]) who were administered chemotherapy plus radiotherapy received treatment at > 60 days (Table 1). 

 Patients aged over 69 years were less likely to receive early treatment (OR = 0.75 [95%CI 0.72–0.78]), whereas those with higher educational levels had a greater chance (OR = 1.58 [95%CI 1.50–1.65]). Patients living in the North region had the lowest probability of receiving treatment within 30 days (OR = 0.49 [95%CI 0.46–0.53]), followed by those in the Midwest (OR = 0.59 [95%CI 0.54–0.64]). Undergoing radiotherapy was associated with an 81% reduction in the chance of receiving treatment within 30 days (OR = 0.19 [95%CI 0.17–0.21]) (Figure 1; Supplementary File 1 — Table 4). 

 Time to treatment initiation for CRC in Brazil averaged 68 days. The shortest intervals wereobserved in Tocantins (42 days), Paraná and Rio Grande do Norte (both 53 days), while thelongest were observed in northern states: Pará (107 days), Acre (92 days) and Amazonas(88 days) (Figure 2; Supplementary File 1 — Table 5). Patients who underwent surgery hadthe shortest intervals, with six states showing a mean interval of approximately 35 days(Rio Grande do Norte, Tocantins, Sergipe, Maranhão, Distrito Federal and Mato Grossodo Sul). Radiotherapy had a longer delay (radiotherapy > 100 days: Roraima, Amapá, Pará,Distrito Federal, Rio de Janeiro, Sergipe, Amazonas and Piauí) (Figure 3; Supplementary File 1 — Table 6). 

 The cumulative probability of treatment initiation at 30 and 60 days was higher among individuals at younger ages (30 and 60%, respectively). Individuals with more than 12 years of education had a 59% probability of treatment initiation at 60 days, compared to only 46% among those with less than ten years. Clinical stage IV showed higher cumulative probabilities (56% at 60 days) compared to stage I (45%) (Figures 4 and 5; Supplementary File 1 — Table 7). 

## DISCUSSION

 The results reveal inequalities in time-to-treatment within 30 days among regions and states of Brazil. The study highlights regional disparities in time to initial treatment for cancer in the country, where the North and Northeast were found to have the worst outcomes in terms of timely treatment of more complex cancers that commonly require multiple treatment approaches (surgical and non-surgical). CVC, for which an early screening and diagnosis policy already exists in Brazil, showed the highest number of cases of early treatment for all states. However, CT treatment also proved to be homogenous throughout Brazil. Routine ordering of ultrasound exams of the thyroid gland by gynecologists may be a factor contributing to this result. 

 Delays in cancer treatment are a problem experienced by health systems worldwide^28^. Moreover, delays initiating treatment are associated with worse prognosis and survival of cancer^16^. In the present study, greater regional disparities in treatment initiation delay were found predominantly for CRC, with an estimated 45,630 new cases of the disease in the country^9^. Time from diagnosis to surgical treatment for CRC patients directly and progressively impacts mortality risk with increased waiting times for surgery^15^. 

 The present results showed a lower proportion of CRC treatment within 60 days in the North (53.5%) and Mid-West (49.6%) regions. Malta et al.^29^ found a greater prevalence of colorectal cancer in Brazil among individuals with higher educational level and who held a private health plan, possibly explained by greater access to diagnosis and improved notification of cases. Our analysis revealed that individuals with more than 12 years of education had a markedly higher cumulative probability of treatment initiation within 60 days, 59% for CRC and 46% for CT, compared to only 46 and 25%, respectively, among those with less than ten years of education. 

 During the 2013–2022 period, treatment initiation time averaged 59 days in Brazil. By contrast, Helsper et al.^14^ found a mean time-to-treatment for CRC of 27 days in Holland, roughly half the length of time observed in Brazil. Currently, there is no consensus on differences in treatment delay for CRC between younger and older adults as a prognostic factor^30^. A systematic review revealed greater time intervals between CRC diagnosis and treatment among younger adults^31^. A population-based study in Canada found a different pattern, in which adults aged < 50 years had shorter times between CRC diagnosis and treatment initiation^13^. Notably, recommendations to screen for CRC in all individuals aged ≥ 50 might lead to a situation where this group receives more rapid accurate diagnosis, whereas younger individuals experience delays. 

 CCNS rank among the malignant tumors with the worst prognosis^32^. The primary treatment for most CRC and CCNS is surgery. However, patients with more advanced lesions may also be offered chemotherapy and/or radiotherapy^33^. Treatment of CRC and CCNS that included chemotherapy and radiotherapy often initiated only at > 60 days (60% and 48%, respectively). Delays in surgery, radiotherapy and chemotherapy negatively impact mortality in these patients^27^. Surgery was the second-most-frequent treatment approach for tumors of the CCNS, with most patients receiving treatment within 30 days (79.5%). Early detection of these cancers, coupled with immediate treatment, is essential for better prognosis^34^. 

 The chance of timely treatment for CCNS was higher in the South and Mid-West regions, but lower in the Northeast. A meta-analysis found that a 4-week delay in treatment was associated with increased mortality for all conventional modalities of treating cancer, with longer delays proving more impactful on patient prognosis^28^. This recurrent inversely proportional pattern between treatment delay and mortality in Brazil warrants further investigation and can help identify additional factors such as the presence of other competing causes (comorbidities), besides advanced staging, that may impact patient survival. This phenomenon can be observed in the North and Northeast regions, where access to cancer treatment is slow, as evidenced by the present study. 

 A previous report noted disparities in time to treat thyroid cancer related with socioeconomic status and demographic characteristics^17^. The present study observed delays in treatment for this type of cancer in the Northeast region of the country. Kowkabany et al.^17^ used data from the National Cancer Database and found longer delays in time to treat thyroid cancer in metropolitan regions of the USA. This finding is corroborated by the present study, in as far as the Southeast region is the most developed metropolitan region in Brazil. 

 The regions showing the longest delays in CVC treatment included the North and Northeast, whereas in the South, Southeast and Mid-West regions early treatment was received. The prognosis of CVC is favorable^35^ and mean time to treatment of CVC in Brazil was 40 days, with significant differences between some states. 

 Although the right to universal, equitable, free health care is enshrined in the Brazilian Constitution^5^, health inequities persist in several regions of the country. Gaining a clearer picture of the social and regional inequalities in the country is of utmost importance in the public health setting for promoting actions and programs to help mitigate disparities in cancer care. 

 Another issue which may impact access to timely treatment for cancer is the need for multidisciplinary teams and multimodal treatment. As evidenced in the current work, individuals requiring more complex treatment experience longer wait times in the system and thus have a lower likelihood of receiving early treatment. These shortcomings are particularly evident in regions with lower levels of development, such as the North and Northeast, which have difficulties providing more complex cancer treatment. 

 This study offers a novel contribution by providing a comprehensive, population-based analysis of time-to-treatment initiation for four distinct cancer types in Brazil, using ten years of nationwide data. By comparing tumor types with differing clinical profiles and technological requirements, the study reveals that patients requiring radiotherapy or chemotherapy face substantially longer delays, particularly in the North and Northeast. This epidemiological scenario may provide evidence for expanding radiotherapy and chemotherapy services in regions with limited availability. In addition, establishing a national system to track referrals and monitor treatment timelines could help ensure compliance with the legally mandated 60-day period between diagnosis and treatment initiation. These findings reinforce the importance of using real-world data to guide cancer control policies and monitor regional inequalities in access to treatment. 

## CONCLUSIONS

 Despite the progress made through public health policies, patients requiring more complex and multimodal technologically advanced treatments still experience significant delays in accessing cancer treatment. Radiotherapy and chemotherapy treatments may contribute to delays in treatment initiation in Brazil, particularly in the North and Northeast regions. Ethnicity and educational level are key determinants of timely access to cancer treatment in the country. Targeted interventions and policy efforts are warranted to help develop effective strategies for ensuring more uniform provision and equitable access to cancer care by the population, reducing wait times for treatment, and improving prognosis of cancer patients in the country. 

## Data Availability

Data supporting the findings of this study are publicly accessible through the IntegradorRHC platform of the Brazilian National Cancer Institute:  https://irhc.inca.gov.br/RHCNet/. R scripts used for data processing and statistical analyses are provided in the supplementary material.

## Footnotes

a Supplementary material available from:  https://zenodo.org/records/17752138
