# Supplementary material for: Inequalities in time to cancer treatment initiation over a decade in Brazil
Source: Rev Saude Publica. 2026 Mar 16;60:e12. doi: 10.11606/s1518-8787.2026060006943 (PMC12991401; doi:10.11606/s1518-8787.2026060006943)
Supplement: Supplementary File 1 [file 1518-8787-rsp-60-e12-md1.docx]

**Supplementary File 1**

**Figure 1.** Probability of treatment initiation among patients diagnosed with cervical, colorectal, central nervous system, and thyroid cancers (Cumulative Incidence Function). IntegradorRHC – INCA, Brazil (2013-2022).


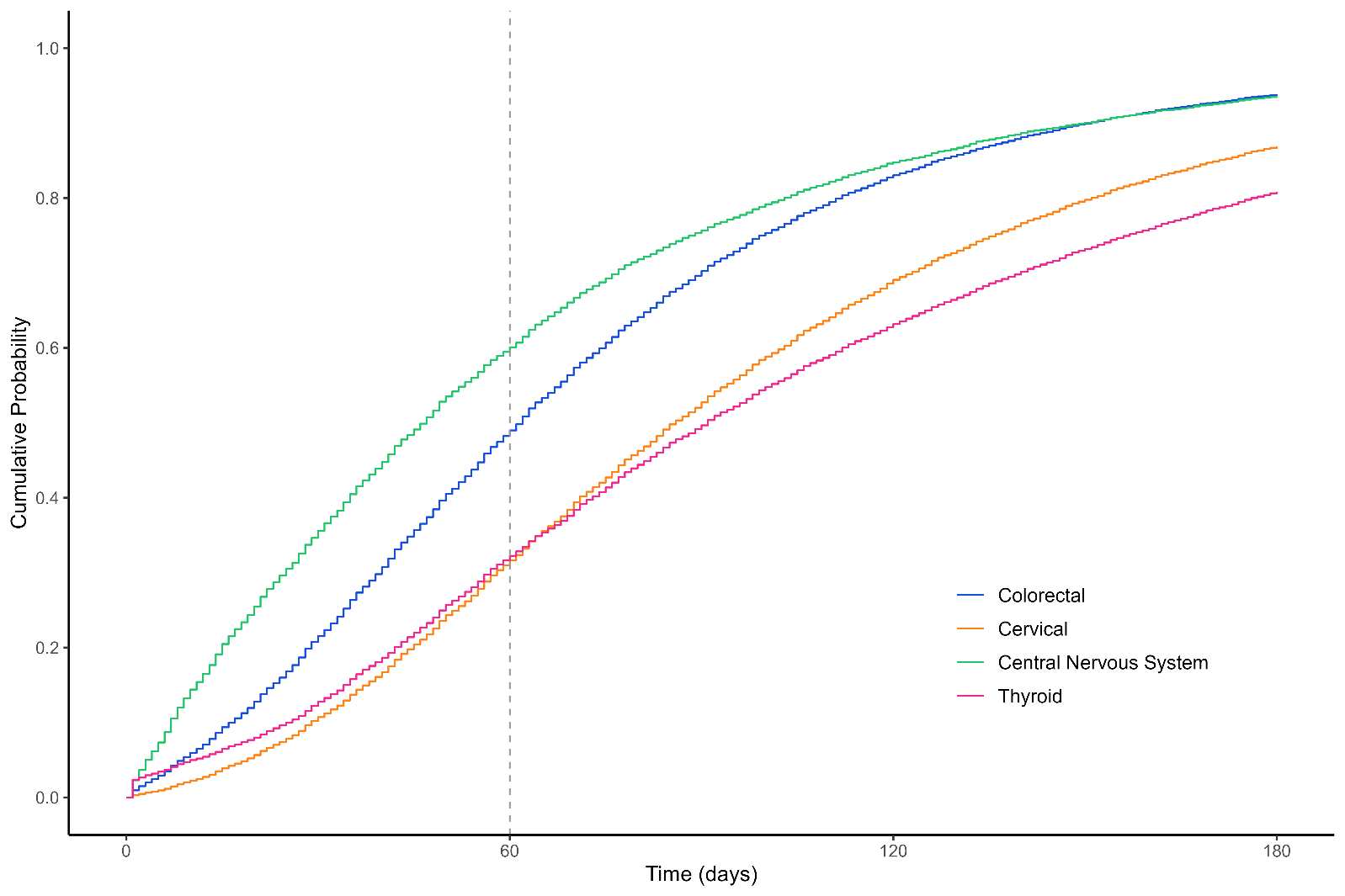


**Table 1.**  Frequency and proportion of missing values among variables in cervix uteri, colorectal, central nervous system, and thyroid cancer cases. IntegradorRHC – INCA, Brazil (2013-2022).

|  | **Missing values** | |
| --- | --- | --- |
| **Variables** | **n = 395,225** | **%** |
| Sex | 0 | 0 |
| Age | 0 | 0 |
| Ethnicity | 124,848 | 31.6 |
| Education | 87,624 | 22.2 |
| Marital status | 38,96 | 9.9 |
| Region of residence | 2,353 | 0.6 |
| Smoking | 204,606 | 51.8 |
| Drinking | 220,070 | 55.7 |
| Clinical stage* | 143,772 | 39.1 |
| Treatment** | 2,525 | 0.7 |

* Clinical staging analysis included cervical (CCX), colorectal (CCR), and thyroid (CT) cancer cases. **Treatment cervical (CCX), colorectal (CCR), and central nervous system (CNS) cancers.

**Table 2.**  Sociodemographic, clinical and habits characteristics of patients with diagnosed of cervix uteri, colorectal, central nervous system, and thyroid cancer. IntegradorRHC – INCA, Brazil (2013-2022).

|  | **CCR** | **CUC** | **CNS** | **CT** |
| --- | --- | --- | --- | --- |
|  | n =168951 (%) | n=141189 (%) | n=27330 (%) | n=57755 (%) |
| **Sex** |  |  |  |  |
| Male | 84926 (50.3) |  | 15158 (55.5) | 10373 (18) |
| Female | 84025 (49.7) | 141189 (100) | 12172 (44.5) | 47382 (82) |
|  |  |  |  |  |
| **Age (years)** |  |  |  |  |
| < 50 | 29319 (17.4) | 87230 (61.8) | 14285 (52.3) | 33596 (58.2) |
| 50-69 | 49323 (29.2) | 11977 (8.5) | 3045 (11.1) | 3860 (6.7) |
| >69 | 90309 (53.5) | 41982 (29.7) | 10000 (36.6) | 20299 (35.1) |
|  |  |  |  |  |
| **Ethnicity** |  |  |  |  |
| White | 57856 (52.7) | 34814 (32.9) | 8279 (47.4) | 13909 (37.4) |
| Black | 5486 (5) | 6582 (6.2) | 753 (4.3) | 1509 (4.1) |
| Brown | 45336 (41.3) | 63134 (59.6) | 8232 (47.2) | 21311 (57.4) |
| Asian | 986 (0.9) | 1050 (1) | 146 (0.8) | 369 (1) |
| Indigenous | 169 (0.2) | 359 (0.3) | 45 (0.3) | 52 (0.1) |
|  |  |  |  |  |
| **Education (years)** |  |  |  |  |
| 0-9 | 90572 (69.2) | 73146 (65.5) | 14469 (68.8) | 20348 (46.4) |
| 10-12 | 27853 (21.3) | 29259 (26.2) | 4612 (21.9) | 13032 (29.7) |
| >12 | 12543 (9.6) | 9317 (8.3) | 1939 (9.2) | 10514 (24) |
|  |  |  |  |  |
| **Marriage status** |  |  |  |  |
| Married | 57452 (57.5) | 42735 (41.7) | 7716 (45.2) | 19289 (53.7) |
| Separated/Viuvo | 20431 (20.5) | 15527 (15.1) | 1705 (10) | 4200 (11.7) |
| Single | 21972 (22) | 44334 (43.2) | 7638 (44.8) | 12438 (34.6) |
|  |  |  |  |  |
| **Region of residence** |  |  |  |  |
| Mid-west | 8206 (4.9) | 7727 (5.5) | 1505 (5.6) | 1531 (2.7) |
| Northeast | 28921 (17.2) | 44888 (32) | 5843 (21.6) | 22608 (39.3) |
| Norht | 6338 (3.8) | 16344 (11.6) | 1771 (6.5) | 2480 (4.3) |
| Southeast | 85298 (50.8) | 48571 (34.6) | 12190 (45) | 22661 (39.4) |
| South | 39130 (23.3) | 22892 (16.3) | 5781 (21.3) | 8187 (14.2) |
|  |  |  |  |  |
| **Smoking** |  |  |  |  |
| Smoker | 10990 (13.6) | 13804 (17.7) | 1146 (11) | 1584 (7.3) |
| Former smoker | 21536 (26.7) | 13130 (16.9) | 2002 (19.2) | 2820 (12.9) |
| Never smoker | 48082 (59.6) | 50874 (65.4) | 7257 (69.7) | 17394 (79.8) |
|  |  |  |  |  |
| **Drinking** |  |  |  |  |
| Drinker | 14882 (19.8) | 11865 (17) | 1615 (16.4) | 2906 (14.3) |
| Former drinker | 12050 (16) | 7746 (11.1) | 1351 (13.7) | 1426 (7) |
| Never drinker | 48416 (64.3) | 50009 (71.8) | 6875 (69.9) | 16014 (78.7) |
|  |  |  |  |  |
| **Clinical stage** |  |  |  |  |
| I | 10913 (9.4) | 14865 (20.2) | * | 24084 (69.8) |
| II | 29907 (25.8) | 22353 (30.3) |  | 3159 (9.2) |
| III | 40116 (34.6) | 26759 (36.3) |  | 4841 (14) |
| IV | 34974 (30.2) | 9747 (13.2) |  | 2405 (7) |
|  |  |  |  |  |
| **Treatment** |  |  |  |  |
| Surgery | 43980 (26.2) | 56020 (40) | 7287 (26.8) | 35697** |
| Surgery plus | 55673 (33.2) | 13737 (9.8) | 6888 (25.4) |  |
| Chemotherapy | 34165 (20.4) | 8450 (6) | 2116 (7.8) |  |
| Radiotherapy | 5632 (3.4) | 13229 (9.4) | 4972 (18.3) |  |
| Radio+Chemotherapy | 14850 (8.9) | 37514 (26.8) | 3062 (11.3) |  |
| Others modalities | 4449 (2.7) | 4725 (3.4) | 825 (3) |  |
| No treatment | 8848 (5.3) | 6516 (4.6) | 2007 (7.4) |  |
|  |  |  |  |  |
| **Time diagnosis-to-treatment (days)** |  |  |  |  |
| 01-30 | 47307 (31.2) | 23929 (18.7) | 12099 (53.7) | 21762 (45.5) |
| 31-60 | 35455 (23.4) | 23342 (18.3) | 3764 (16.7) | 5407 (11.3) |
| >60 | 68789 (45.4) | 80387 (63) | 6660 (29.6) | 20654 (43.2) |

* Clinical stage analysis was not performed in CNS cases due to their distint classification system in relation to solid tumor staging (TNM) . **O tratamento do CT consiste em cirurgia e radioiodoterapia, devivo a falta de clareza em relação ao tratamento de radioioterapia, apenas o tratamento cirúrgico foi considerado para CT.

**Table 3.** Univariate analysis of patients with diagnosed of cervix uteri, colorectal, central nervous system, and thyroid cancer. IntegradorRHC – INCA, Brazil (2013-2022).

|  | **CNS** | | | | | | **CUC** | | | | | | **CT** | | | | | | **CCR** | | | | | |
| --- | --- | --- | --- | --- | --- | --- | --- | --- | --- | --- | --- | --- | --- | --- | --- | --- | --- | --- | --- | --- | --- | --- | --- | --- |
|  | **0-30** | | | **31-60** | | | **0-30** | | | **31-60** | | | **0-30** | | | **31-60** | | | **0-30** | | | **31-60** | | |
|  | **OR** | **CI 95%** |  | **OR** | **CI 95%** |  | **OR** | **CI 95%** |  | **OR** | **CI 95%** |  | **OR** | **CI 95%** |  | **OR** | **CI 95%** |  | **OR** | **CI 95%** |  | **OR** | **CI 95%** |  |
|  |  | **Lower** | **Upper** |  | **Lower** | **Upper** |  | **Lower** | **Upper** |  | **Lower** | **Upper** |  | **Lower** | **Upper** |  | **Lower** | **Upper** |  | **Lower** | **Upper** |  | **Lower** | **Upper** |
| **Sex** |  |  |  |  |  |  |  |  |  |  |  |  |  |  |  |  |  |  |  |  |  |  |  |  |
| Male | 1.03 | 0.97 | 1.10 | 1.06 | 0.98 | 1.15 |  |  |  |  |  |  |  |  |  |  |  |  | 0.98 | 0.96 | 1.01 | 1.06 | 1.03 | 1.08 |
| Female | 1.00 |  |  | 1.00 |  |  |  |  |  |  |  |  | 1.00 |  |  | 1.00 |  |  | 1.00 |  |  | 1.00 |  |  |
|  |  |  |  |  |  |  |  |  |  |  |  |  | 1.10 | 1.05 | 1.16 | 1.32 | 1.22 | 1.42 |  |  |  |  |  |  |
| **Age (years)** |  |  |  |  |  |  |  |  |  |  |  |  |  |  |  |  |  |  |  |  |  |  |  |  |
| < 50 | 1.00 |  |  | 1.00 |  |  | 1.00 |  |  | 1.00 |  |  | 1.00 |  |  | 1.00 |  |  | 1.00 |  |  | 1.00 |  |  |
| 50-69 | 1.11 | 1.04 | 1.18 | 1.34 | 1.23 | 1.46 | 0.78 | 0.76 | 0.81 | 0.99 | 0.96 | 1.02 | 0.96 | 0.92 | 0.99 | 0.71 | 0.66 | 0.76 | 0.79 | 0.76 | 0.81 | 0.82 | 0.79 | 0.85 |
| >69 | 1.47 | 1.32 | 1.63 | 1.49 | 1.29 | 1.72 | 0.88 | 0.83 | 0.93 | 1.13 | 1.07 | 1.19 | 0.85 | 0.79 | 0.92 | 0.69 | 0.60 | 0.79 | 0.81 | 0.78 | 0.84 | 0.74 | 0.71 | 0.77 |
|  |  |  |  |  |  |  |  |  |  |  |  |  |  |  |  |  |  |  |  |  |  |  |  |  |
| **Education (years)** |  |  |  |  |  |  |  |  |  |  |  |  |  |  |  |  |  |  |  |  |  |  |  |  |
| 0-9 | 1.00 |  |  | 1.00 |  |  | 1.00 |  |  | 1.00 |  |  | 1.00 |  |  | 1.00 |  |  | 1.00 |  |  | 1.00 |  |  |
| 10-12 | 0.75 | 0.69 | 0.69 | 0.83 | 0.75 | 0.93 | 1.13 | 1.09 | 1.17 | 1.04 | 1.00 | 1.08 | 1.08 | 1.02 | 1.13 | 1.29 | 1.19 | 1.41 | 1.19 | 1.15 | 1.23 | 1.20 | 1.16 | 1.24 |
| >12 | 0.88 | 0.78 | 0.99 | 0.93 | 0.79 | 1.09 | 1.60 | 1.51 | 1.69 | 1.38 | 1.30 | 1.46 | 1.83 | 1.73 | 1.94 | 2.84 | 2.60 | 3.09 | 1.63 | 1.55 | 1.70 | 1.48 | 1.41 | 1.56 |
|  |  |  |  |  |  |  |  |  |  |  |  |  |  |  |  |  |  |  |  |  |  |  |  |  |
| **Marital status** |  |  |  |  |  |  |  |  |  |  |  |  |  |  |  |  |  |  |  |  |  |  |  |  |
| Married | 0.82 | 0.76 | 0.89 | 1.10 | 1.00 | 1.22 | 1.14 | 1.10 | 1.18 | 1.11 | 1.07 | 1.15 | 1.07 | 1.01 | 1.13 | 1.24 | 1.13 | 1.36 | 1.17 | 1.12 | 1.22 | 1.18 | 1.13 | 1.23 |
| Separated/Widowed | 0.99 | 0.86 | 1.13 | 1.16 | 0.98 | 1.37 | 1.01 | 0.95 | 1.06 | 1.11 | 1.05 | 1.16 | 1.03 | 0.94 | 1.12 | 1.22 | 1.06 | 1.41 | 1.05 | 1.00 | 1.10 | 1.00 | 0.95 | 1.05 |
| Single | 1.00 |  |  | 1.00 |  |  | 1.00 |  |  | 1.00 |  |  | 1.00 |  |  | 1.00 |  |  | 1.00 |  |  | 1.00 |  |  |
|  |  |  |  |  |  |  |  |  |  |  |  |  |  |  |  |  |  |  |  |  |  |  |  |  |
| **Region of residence** |  |  |  |  |  |  |  |  |  |  |  |  |  |  |  |  |  |  |  |  |  |  |  |  |
| Mid-west | 1.24 | 1.07 | 1.43 | 0.78 | 0.64 | 0.95 | 0.885 | 0.79 | 0.91 | 0.65 | 0.61 | 0.71 | 0.43 | 0.38 | 0.49 | 0.72 | 0.58 | 0.90 | 0.67 | 0.63 | 0.71 | 0.71 | 0.66 | 0.75 |
| Northeast | 0.50 | 0.46 | 0.55 | 0.57 | 0.51 | 0.63 | 0.60 | 0.57 | 0.62 | 0.78 | 0.75 | 0.82 | 0.41 | 0.38 | 0.43 | 0.67 | 0.60 | 0.75 | 0.71 | 0.69 | 0.74 | 0.84 | 0.91 | 0.98 |
| Norht | 0.67 | 0.58 | 0.76 | 0.58 | 0.49 | 0.69 | 0.36 | 0.34 | 0.38 | 0.43 | 0.41 | 0.46 | 0.20 | 0.17 | 0.22 | 0.50 | 0.41 | 0.60 | 0.56 | 0.52 | 0.60 | 0.62 | 0.58 | 0.67 |
| Southeast | 1.65 | 1.52 | 1.80 | 0.71 | 0.64 | 0.79 | 0.63 | 0.61 | 0.66 | 0.59 | 0.56 | 0.61 | 0.50 | 0.47 | 0.53 | 1.22 | 1.09 | 1.35 | 0.76 | 0.74 | 0.78 | 0.78 | 0.75 | 0.80 |
| South | 1.00 |  |  | 1.00 |  |  | 1.00 |  |  | 1.00 |  |  | 1.00 |  |  | 1.00 |  |  | 1.00 |  |  | 1.00 |  |  |
|  |  |  |  |  |  |  |  |  |  |  |  |  |  |  |  |  |  |  |  |  |  |  |  |  |
| **Treatment** |  |  |  |  |  |  |  |  |  |  |  |  |  |  |  |  |  |  |  |  |  |  |  |  |
| Surgery | 1.00 |  |  | 1.00 |  |  | 1.00 |  |  | 1.00 |  |  |  |  |  |  |  |  | 1.00 |  |  | 1.00 |  |  |
| Surgery plus | 0.94 | 0.84 | 1.05 | 0.94 | 0.80 | 1.11 | 1.15 | 1.10 | 1.21 | 1.72 | 1.64 | 1.82 |  |  |  |  |  |  | 0.68 | 0.66 | 0.70 | 1.15 | 1.11 | 1.19 |
| Chemotherapy | 0.12 | 0.11 | 0.14 | 0.68 | 0.58 | 0.81 | 0.67 | 0.63 | 0.72 | 1.91 | 1.81 | 2.03 |  |  |  |  |  |  | 0.32 | 0.31 | 0.33 | 1.25 | 1.21 | 1.30 |
| Radiotherapy | 0.05 | 0.05 | 0.06 | 0.69 | 0.61 | 0.79 | 0.40 | 0.37 | 0.42 | 1.20 | 1.14 | 1.26 |  |  |  |  |  |  | 0.19 | 0.17 | 0.20 | 0.86 | 0.80 | 0.92 |
| Radio+Chemotherapy | 0.07 | 0.06 | 0.07 | 0.84 | 0.73 | 0.97 | 0.45 | 0.43 | 0.47 | 1.66 | 1.60 | 1.72 |  |  |  |  |  |  | 0.19 | 0.18 | 0.20 | 0.96 | 0.92 | 1.01 |

**Table 4.** Multiple models of patients with diagnosed of cervix uteri, colorectal, central nervous system, and thyroid cancer. IntegradorRHC – INCA, Brazil (2013-2022).

|  | **CNS** | | | | | | **CUC** | | | | | | **CT** | | | | | | **CCR** | | | | | |
| --- | --- | --- | --- | --- | --- | --- | --- | --- | --- | --- | --- | --- | --- | --- | --- | --- | --- | --- | --- | --- | --- | --- | --- | --- |
|  | **0-30** | | | **31-60** | | | **0-30** | | | **31-60** | | | **0-30** | | | **31-60** | | | **0-30** | | | **31-60** | | |
|  | **OR** | **CI 95%** |  | **OR** | **CI 95%** |  | **OR** | **CI 95%** |  | **OR** | **CI 95%** |  | **OR** | **CI 95%** |  | **OR** | **CI 95%** |  | **OR** | **CI 95%** |  | **OR** | **CI 95%** |  |
|  |  | **Lower** | **Upper** |  | **Lower** | **Upper** |  | **Lower** | **Upper** |  | **Lower** | **Upper** |  | **Lower** | **Upper** |  | **Lower** | **Upper** |  | **Lower** | **Upper** |  | **Lower** | **Upper** |
| **Sex** |  |  |  |  |  |  |  |  |  |  |  |  |  |  |  |  |  |  |  |  |  |  |  |  |
| Male | 1.15 | 1.02 | 1.29 | 1.2 | 1.06 | 1.36 |  |  |  |  |  |  |  |  |  |  |  |  | 1.01 | 0.98 | 1.04 | 1.06 | 1.03 | 1.09 |
| Female | 1.00 |  |  | 1.00 |  |  |  |  |  |  |  |  | 1.00 |  |  | 1.00 |  |  | 1.00 |  |  | 1.00 |  |  |
|  |  |  |  |  |  |  |  |  |  |  |  |  | 1.06 | 1,00 | 1.13 | 1.26 | 1.15 | 1.38 |  |  |  |  |  |  |
| **Age (years)** |  |  |  |  |  |  |  |  |  |  |  |  |  |  |  |  |  |  |  |  |  |  |  |  |
| < 50 | 1.00 |  |  | 1.00 |  |  | 1.00 |  |  | 1.00 |  |  | 1.00 |  |  | 1.00 |  |  | 1.00 |  |  | 1.00 |  |  |
| 50-69 | 0.97 | 0.85 | 1.09 | 1.28 | 1.11 | 1.46 | 0.96 | 0.92 | 1,00 | 0.89 | 0.86 | 0.93 | 1.02 | 0.97 | 1.08 | 0.8 | 0.73 | 0.87 | 0.75 | 0.72 | 0.78 | 0.84 | 0.81 | 0.88 |
| >69 | 1.21 | 0.98 | 1.49 | 1.22 | 0.97 | 1.54 | 1.25 | 1.17 | 1.34 | 1.06 | 1,00 | 1.13 | 0.94 | 0.85 | 1.04 | 0.98 | 0.84 | 1.15 | 0.75 | 0.72 | 0.78 | 0.8 | 0.76 | 0.83 |
|  |  |  |  |  |  |  |  |  |  |  |  |  |  |  |  |  |  |  |  |  |  |  |  |  |
| **Education (years)** |  |  |  |  |  |  |  |  |  |  |  |  |  |  |  |  |  |  |  |  |  |  |  |  |
| 0-9 | 1.00 |  |  | 1.00 |  |  | 1.00 |  |  | 1.00 |  |  | 1.00 |  |  | 1.00 |  |  | 1.00 |  |  | 1.00 |  |  |
| 10-12 | 0.7 | 0.61 | 0.8 | 0.79 | 0.68 | 0.92 | 1.04 | 1,00 | 1.08 | 1.12 | 1.08 | 1.17 | 1.15 | 1.08 | 1.21 | 1.3 | 1.18 | 1.43 | 1.17 | 1.13 | 1.18 | 1.13 | 1.22 | 1.24 |
| >12 | 0.76 | 0.63 | 0.93 | 0.9 | 0.73 | 1.11 | 1.42 | 1.34 | 1.51 | 1.53 | 1.44 | 1.63 | 2.09 | 1.96 | 2.22 | 2.74 | 2.49 | 3.02 | 1.58 | 1.5 | 1.5 | 1.43 | 1.58 | 1.56 |
|  |  |  |  |  |  |  |  |  |  |  |  |  |  |  |  |  |  |  |  |  |  |  |  |  |
| **Marital status** |  |  |  |  |  |  |  |  |  |  |  |  |  |  |  |  |  |  |  |  |  |  |  |  |
| Married |  |  |  |  |  |  |  |  |  |  |  |  |  |  |  |  |  |  |  |  |  |  |  |  |
| Separated/Widowed |  |  |  |  |  |  |  |  |  |  |  |  |  |  |  |  |  |  |  |  |  |  |  |  |
| Single |  |  |  |  |  |  |  |  |  |  |  |  |  |  |  |  |  |  |  |  |  |  |  |  |
|  |  |  |  |  |  |  |  |  |  |  |  |  |  |  |  |  |  |  |  |  |  |  |  |  |
| **Region of residence** |  |  |  |  |  |  |  |  |  |  |  |  |  |  |  |  |  |  |  |  |  |  |  |  |
| Mid-west | 0.62 | 0.47 | 0.82 | 0.71 | 0.51 | 0.97 | 0.73 | 0.66 | 0.82 | 0.57 | 0.51 | 0.64 | 0.65 | 0.55 | 0.77 | 0.63 | 0.46 | 0.88 | 0.59 | 0.54 | 0.64 | 0.64 | 0.59 | 0.71 |
| Northeast | 0.57 | 0.49 | 0.67 | 0.54 | 0.46 | 0.64 | 0.65 | 0.62 | 0.68 | 0.74 | 0.7 | 0.77 | 0.55 | 0.51 | 0.59 | 0.74 | 0.65 | 0.84 | 0.76 | 0.73 | 0.79 | 0.96 | 0.92 | 1.01 |
| Norht | 0.34 | 0.28 | 0.42 | 0.5 | 0.4 | 0.62 | 0.3 | 0.28 | 0.32 | 0.36 | 0.34 | 0.39 | 0.17 | 0.15 | 0.19 | 0.36 | 0.28 | 0.45 | 0.49 | 0.46 | 0.53 | 0.54 | 0.5 | 0.59 |
| Southeast | 0.82 | 0.7 | 0.96 | 0.66 | 0.56 | 0.78 | 0.56 | 0.53 | 0.58 | 0.56 | 0.54 | 0.59 | 0.41 | 0.38 | 0.44 | 0.97 | 0.87 | 1.09 | 0.63 | 0.61 | 0.65 | 0.76 | 0.74 | 0.79 |
| South | 1.00 |  |  | 1.00 |  |  | 1.00 |  |  | 1.00 |  |  | 1.00 |  |  | 1.00 |  |  | 1.00 |  |  | 1.00 |  |  |
|  |  |  |  |  |  |  |  |  |  |  |  |  |  |  |  |  |  |  |  |  |  |  |  |  |
| **Treatment** |  |  |  |  |  |  |  |  |  |  |  |  |  |  |  |  |  |  |  |  |  |  |  |  |
| Surgery | 1.00 |  |  | 1.00 |  |  | 1.00 |  |  | 1.00 |  |  |  |  |  |  |  |  | 1.00 |  |  | 1.00 |  |  |
| Surgery plus | 0.91 | 0.76 | 1.09 | 0.94 | 0.73 | 1.23 | 1.26 | 1.19 | 1.32 | 1.8 | 1.7 | 1.91 |  |  |  |  |  |  | 0.71 | 0.69 | 0.74 | 1.16 | 1.12 | 1.21 |
| Chemotherapy | 0.15 | 0.12 | 0.18 | 0.71 | 0.55 | 0.91 | 0.71 | 0.66 | 0.77 | 2.06 | 1.92 | 2.21 |  |  |  |  |  |  | 0.33 | 0.31 | 0.34 | 1.25 | 1.2 | 1.31 |
| Radiotherapy | 0.06 | 0.05 | 0.07 | 0.72 | 0.59 | 0.89 | 0.42 | 0.39 | 0.45 | 1.25 | 1.17 | 1.33 |  |  |  |  |  |  | 0.19 | 0.17 | 0.21 | 0.86 | 0.8 | 0.94 |
| Radio+Chemotherapy | 0.07 | 0.06 | 0.09 | 0.76 | 0.61 | 0.95 | 0.5 | 0.48 | 0.53 | 1.77 | 1.7 | 1.84 |  |  |  |  |  |  | 0.21 | 0.2 | 0.22 | 0.98 | 0.93 | 1.03 |

**Table 5.** Mean time in days between diagnosis and treatment of cases of cervix uteri, colorectal, central nervous system, and thyroid cancer by state in Brazil . IntegradorRHC – INCA, Brazil (2013-2022).

| **Brazilian state** | **Mean time (days)** | | | |
| --- | --- | --- | --- | --- |
|  | **CNS** | **CUC** | **CT** | **CCR** |
| Acre | 96,6 | 120,1 | 138,1 | 91,9 |
| Alagoas | 58,4 | 100,5 | 111,5 | 71,7 |
| Amazonas | 56,7 | 152,8 | 82,3 | 88,1 |
| Amapá | 48,7 | 101,7 | 118,1 | 72,0 |
| Bahia | 76,1 | 99,5 | 72,5 | 78,4 |
| Ceará | 51,1 | 72,9 | 85,0 | 66,3 |
| Distrito Federal | 43,6 | 72,8 | 100,0 | 68,5 |
| Espírito Santo | 65,2 | 83,1 | 72,4 | 56,7 |
| Goiás | 36,4 | 87,5 | 75,7 | 85,1 |
| Maranhão | 89,8 | 102,0 | 39,5 | 66,0 |
| Minas Gerais | 29,9 | 86,8 | 54,6 | 60,7 |
| Mato Grosso do Sul | 52,1 | 88,0 | 60,1 | 58,3 |
| Mato Grosso | 61,2 | 113,5 | 95,8 | 76,0 |
| Para | 83,8 | 129,9 | 168,8 | 106,9 |
| Paraíba | 82,6 | 109,5 | 43,0 | 90,6 |
| Pernambuco | 80,8 | 100,8 | 88,6 | 64,3 |
| Piauí | 55,8 | 89,4 | 59,1 | 73,6 |
| Paraná | 38,5 | 65,4 | 42,4 | 52,4 |
| Rio de Janeiro | 75,6 | 129,2 | 115,1 | 89,8 |
| Rio Grande do Norte | 52,5 | 58,6 | 41,6 | 53,0 |
| Rondônia | 64,5 | 92,5 | 95,8 | 80,2 |
| Roraima | 62,6 | 164,4 | 97,2 | 73,6 |
| Rio Grande do Sul | 38,3 | 81,4 | 32,7 | 64,4 |
| Santa Catarina | 65,9 | 108,1 | 47,3 | 77,6 |
| Sergipe | 81,9 | 117,2 | 123,2 | 78,4 |
| São Paulo | 32,1 | 96,4 | 69,1 | 68,5 |
| Tocantins | 20,5 | 59,0 | 33,2 | 41,7 |

**Table 6.** Mean time in days between diagnosis and treatment of cases of cervix uteri, colorectal, central nervous system, and thyroid cancer by treatment modality and state in Brazil . IntegradorRHC – INCA, Brazil (2013-2022).

|  | **Surgery** | | | |  | **Surgery plus** | | | |  | **Radiotherapy** | | | |  | **Chemotherapy** | | | |  | **Radio + Chemotheraphy** | | | |
| --- | --- | --- | --- | --- | --- | --- | --- | --- | --- | --- | --- | --- | --- | --- | --- | --- | --- | --- | --- | --- | --- | --- | --- | --- |
| **Brazilian state** | **Mean time (days)** | | | |  | **Mean time (days)** | | | |  | **Mean time (days)** | | | |  | **Mean time (days)** | | | |  | **Mean time (days)** | | | |
|  | **CNS** | **CUC** | **CT** | **CCR** |  | **CNS** | **CUC** | **CT** | **CCR** |  | **CNS** | **CUC** | **CT** | **CCR** |  | **CNS** | **CUC** | **CT** | **CCR** |  | **CNS** | **CUC** | **CT** | **CCR** |
| Acre | 15,9 | 137,5 | 118,0 | 87,2 |  | 72,8 | 90,6 |  | 77,4 |  | 83,1 | 84,0 |  | 71,5 |  | 111,3 | 115,4 |  | 81,0 |  | 71,9 | 89,1 |  | 81,0 |
| Alagoas | 22,9 | 100,7 | 74,1 | 50,0 |  | 25,8 | 71,7 |  | 62,8 |  | 89,8 | 89,2 |  | 85,8 |  | 62,4 | 95,9 |  | 66,3 |  | 75,8 | 86,5 |  | 81,5 |
| Amazonas | 24,2 | 126,2 | 60,9 | 61,6 |  | 27,6 | 98,8 |  | 65,3 |  | 106,3 | 125,6 |  | 104,7 |  | 81,9 | 121,7 |  | 94,7 |  | 89,1 | 118,1 |  | 93,0 |
| Amapá | 15,9 | 85,7 | 58,5 | 59,0 |  | 24,3 | 62,7 |  | 55,7 |  | 62,4 | 100,1 |  | 127,5 |  | 42,3 | 109,1 |  | 48,1 |  | 109,1 | 96,0 |  | 86,0 |
| Bahia | 21,9 | 70,4 | 38,2 | 57,0 |  | 19,8 | 86,3 |  | 58,8 |  | 96,5 | 119,3 |  | 89,3 |  | 83,1 | 94,1 |  | 81,5 |  | 83,8 | 93,0 |  | 78,1 |
| Ceará | 23,2 | 78,5 | 70,5 | 53,2 |  | 37,1 | 63,2 |  | 58,7 |  | 70,3 | 71,3 |  | 69,1 |  | 42,1 | 69,7 |  | 72,5 |  | 61,8 | 61,1 |  | 61,5 |
| Distrito Federal | 25,2 | 53,8 | 82,8 | 33,8 |  | 21,8 | 77,2 |  | 64,6 |  | 96,2 | 100,6 |  | 112,2 |  | 78,7 | 91,3 |  | 96,9 |  | 58,1 | 89,5 |  | 105,5 |
| Espírito Santo | 10,6 | 69,6 | 55,8 | 43,1 |  | 28,4 | 70,4 |  | 50,0 |  | 91,2 | 92,5 |  | 90,1 |  | 48,8 | 80,1 |  | 58,6 |  | 86,4 | 81,5 |  | 69,0 |
| Goiás | 22,3 | 71,9 | 63,2 | 63,6 |  | 13,1 | 86,4 |  | 95,1 |  | 58,7 | 80,5 |  | 84,6 |  | 86,4 | 79,9 |  | 75,7 |  | 61,6 | 97,3 |  | 101,2 |
| Maranhão | 27,6 | 71,8 | 28,6 | 34,5 |  | 17,7 | 77,9 |  | 49,7 |  | 102,1 | 105,7 |  | 98,9 |  | 91,5 | 98,6 |  | 75,1 |  | 98,2 | 107,3 |  | 85,7 |
| Minas Gerais | 15,2 | 85,4 | 50,0 | 51,1 |  | 12,1 | 72,7 |  | 51,0 |  | 64,7 | 82,8 |  | 73,6 |  | 65,6 | 79,6 |  | 69,6 |  | 60,4 | 77,4 |  | 74,7 |
| Mato Grosso do Sul | 21,6 | 72,7 | 49,0 | 35,0 |  | 23,6 | 64,5 |  | 45,4 |  | 71,8 | 102,1 |  | 82,4 |  | 76,2 | 75,9 |  | 73,8 |  | 51,8 | 82,8 |  | 72,9 |
| Mato Grosso | 23,6 | 106,4 | 77,8 | 65,8 |  | 13,3 | 87,5 |  | 72,4 |  | 74,4 | 103,1 |  | 65,7 |  | 64,0 | 74,8 |  | 67,3 |  | 68,0 | 86,8 |  | 68,4 |
| Para | 34,2 | 122,1 | 92,7 | 87,9 |  | 40,2 | 109,1 |  | 80,8 |  | 104,3 | 125,2 |  | 121,9 |  | 89,3 | 103,5 |  | 91,6 |  | 94,2 | 101,7 |  | 94,2 |
| Paraíba | 34,0 | 65,8 | 30,6 | 48,5 |  | 38,0 | 76,8 |  | 66,4 |  | 78,4 | 121,4 |  | 89,0 |  | 78,0 | 106,4 |  | 93,5 |  | 63,7 | 89,7 |  | 86,4 |
| Pernambuco | 9,6 | 88,0 | 74,9 | 42,2 |  | 28,5 | 83,4 |  | 46,4 |  | 100,7 | 99,9 |  | 87,6 |  | 79,3 | 84,7 |  | 69,9 |  | 82,5 | 89,2 |  | 76,9 |
| Piauí | 14,6 | 76,4 | 44,4 | 54,0 |  | 22,0 | 80,9 |  | 60,4 |  | 92,6 | 91,1 |  | 100,8 |  | 69,2 | 70,3 |  | 74,5 |  | 59,1 | 86,0 |  | 82,7 |
| Paraná | 11,3 | 59,7 | 31,8 | 41,0 |  | 14,1 | 41,5 |  | 41,9 |  | 61,7 | 85,8 |  | 75,3 |  | 78,0 | 65,7 |  | 64,3 |  | 49,4 | 64,4 |  | 57,4 |
| Rio de Janeiro | 31,4 | 114,8 | 70,6 | 65,5 |  | 38,2 | 94,3 |  | 70,3 |  | 90,7 | 112,5 |  | 108,4 |  | 105,9 | 105,7 |  | 96,3 |  | 91,7 | 113,7 |  | 106,5 |
| Rio Grande do Norte | 13,6 | 42,7 | 25,7 | 32,0 |  | 21,2 | 48,2 |  | 39,8 |  | 61,7 | 77,9 |  | 77,8 |  | 57,9 | 65,5 |  | 59,4 |  | 65,9 | 65,8 |  | 78,3 |
| Rondônia | 46,3 | 91,6 | 69,3 | 75,9 |  | 38,8 | 75,6 |  | 77,3 |  | 50,9 | 79,9 |  | 68,4 |  | 111,2 | 90,5 |  | 67,6 |  | 60,1 | 89,9 |  | 83,1 |
| Roraima | 20,5 | 136,9 | 60,5 | 42,8 |  | 19,3 | 98,0 |  | 45,8 |  | 45,3 | 108,1 |  | 167,0 |  | 180,0 | 97,4 |  | 68,6 |  | 130,0 | 138,2 |  | 115,3 |
| Rio Grande do Sul | 10,1 | 66,7 | 24,6 | 38,6 |  | 10,2 | 51,2 |  | 44,8 |  | 68,7 | 87,7 |  | 91,6 |  | 61,9 | 75,7 |  | 68,3 |  | 60,8 | 76,0 |  | 72,3 |
| Santa Catarina | 16,3 | 103,7 | 31,4 | 48,8 |  | 53,8 | 82,6 |  | 70,9 |  | 78,1 | 93,1 |  | 92,0 |  | 73,2 | 84,1 |  | 77,8 |  | 63,7 | 90,6 |  | 89,3 |
| Sergipe | 14,0 | 68,2 | 89,6 | 34,7 |  | 48,5 | 93,1 |  | 74,1 |  | 89,3 | 120,9 |  | 106,6 |  | 64,6 | 97,3 |  | 81,2 |  | 80,6 | 106,9 |  | 94,5 |
| São Paulo | 17,0 | 83,9 | 58,0 | 51,8 |  | 17,3 | 78,6 |  | 61,7 |  | 70,2 | 95,8 |  | 92,2 |  | 51,3 | 82,7 |  | 72,2 |  | 59,7 | 86,9 |  | 86,6 |
| Tocantins | 5,4 | 22,2 | 34,6 | 34,3 |  | 13,2 | 52,5 |  | 30,6 |  | 30,3 | 76,0 |  | 87,5 |  | 59,3 | 99,3 |  | 61,2 |  | 49,6 | 84,0 |  | 94,5 |

**Table 7.** Mean time in days between diagnosis and treatment of cases of cervix uteri, colorectal, central nervous system, and thyroid cancer by treatment modality and state in Brazil . IntegradorRHC – INCA, Brazil (2013-2022).

|  | **Time (days)** | | | | | | | | | | | | | | | | | | | | | | | |
| --- | --- | --- | --- | --- | --- | --- | --- | --- | --- | --- | --- | --- | --- | --- | --- | --- | --- | --- | --- | --- | --- | --- | --- | --- |
|  | All cancers | | | |  | Central Nervous System Cancer | | | |  | Cervical Cancer | | | |  | Thyroid Cancer | | | |  | Colorectal Cancer | | | |
|  | **30** | **60** | **90** | **180** |  | **30** | **60** | **90** | **180** |  | **30** | **60** | **90** | **180** |  | **30** | **60** | **90** | **180** |  | **30** | **60** | **90** | **180** |
| **Sex** |  |  |  |  |  |  |  |  |  |  |  |  |  |  |  |  |  |  |  |  |  |  |  |  |
| Male | 0,22 | 0,49 | 0,70 | 0,93 |  | 0,35 | 0,60 | 0,76 | 0,94 |  |  |  |  |  |  | 0,15 | 0,37 | 0,56 | 0,84 |  | 0,21 | 0,49 | 0,70 | 0,94 |
| Female | 0,14 | 0,37 | 0,58 | 0,88 |  | 0,34 | 0,59 | 0,75 | 0,93 |  | 0,10 | 0,31 | 0,52 | 0,87 |  | 0,12 | 0,30 | 0,48 | 0,80 |  | 0,21 | 0,48 | 0,69 | 0,94 |
|  |  |  |  |  |  |  |  |  |  |  |  |  |  |  |  |  |  |  |  |  |  |  |  |  |
| **Age (years)** |  |  |  |  |  |  |  |  |  |  |  |  |  |  |  |  |  |  |  |  |  |  |  |  |
| < 35 | 0,17 | 0,39 | 0,59 | 0,88 |  | 0,41 | 0,64 | 0,77 | 0,93 |  | 0,10 | 0,31 | 0,53 | 0,87 |  | 0,13 | 0,36 | 0,55 | 0,84 |  | 0,30 | 0,60 | 0,79 | 0,95 |
| 35-49 | 0,14 | 0,37 | 0,57 | 0,88 |  | 0,26 | 0,48 | 0,67 | 0,91 |  | 0,10 | 0,30 | 0,51 | 0,86 |  | 0,12 | 0,32 | 0,50 | 0,81 |  | 0,23 | 0,53 | 0,74 | 0,95 |
| 50-69 | 0,17 | 0,42 | 0,63 | 0,91 |  | 0,32 | 0,60 | 0,77 | 0,95 |  | 0,10 | 0,31 | 0,51 | 0,87 |  | 0,12 | 0,29 | 0,45 | 0,78 |  | 0,20 | 0,48 | 0,69 | 0,94 |
| > 69 | 0,19 | 0,43 | 0,64 | 0,92 |  | 0,38 | 0,65 | 0,83 | 0,96 |  | 0,12 | 0,34 | 0,55 | 0,89 |  | 0,14 | 0,30 | 0,46 | 0,77 |  | 0,20 | 0,46 | 0,67 | 0,93 |
|  |  |  |  |  |  |  |  |  |  |  |  |  |  |  |  |  |  |  |  |  |  |  |  |  |
| **Ethnicity** |  |  |  |  |  |  |  |  |  |  |  |  |  |  |  |  |  |  |  |  |  |  |  |  |
| White | 0,19 | 0,46 | 0,67 | 0,92 |  | 0,31 | 0,60 | 0,78 | 0,95 |  | 0,13 | 0,37 | 0,58 | 0,90 |  | 0,13 | 0,32 | 0,50 | 0,83 |  | 0,23 | 0,52 | 0,73 | 0,95 |
| Black | 0,14 | 0,36 | 0,56 | 0,88 |  | 0,25 | 0,49 | 0,64 | 0,89 |  | 0,10 | 0,29 | 0,49 | 0,85 |  | 0,11 | 0,21 | 0,36 | 0,73 |  | 0,19 | 0,45 | 0,66 | 0,92 |
| Brown | 0,14 | 0,37 | 0,57 | 0,88 |  | 0,30 | 0,53 | 0,70 | 0,92 |  | 0,10 | 0,30 | 0,50 | 0,85 |  | 0,10 | 0,25 | 0,42 | 0,77 |  | 0,20 | 0,48 | 0,70 | 0,93 |
|  |  |  |  |  |  |  |  |  |  |  |  |  |  |  |  |  |  |  |  |  |  |  |  |  |
| **Education (years)** |  |  |  |  |  |  |  |  |  |  |  |  |  |  |  |  |  |  |  |  |  |  |  |  |
| < 10 | 0,16 | 0,39 | 0,60 | 0,90 |  | 0,37 | 0,62 | 0,77 | 0,94 |  | 0,10 | 0,30 | 0,51 | 0,87 |  | 0,11 | 0,25 | 0,40 | 0,76 |  | 0,20 | 0,46 | 0,68 | 0,94 |
| 10-12 | 0,16 | 0,40 | 0,61 | 0,89 |  | 0,29 | 0,53 | 0,70 | 0,92 |  | 0,10 | 0,31 | 0,52 | 0,87 |  | 0,10 | 0,27 | 0,46 | 0,79 |  | 0,22 | 0,51 | 0,73 | 0,94 |
| >12 | 0,21 | 0,49 | 0,69 | 0,92 |  | 0,33 | 0,58 | 0,76 | 0,94 |  | 0,13 | 0,38 | 0,60 | 0,90 |  | 0,19 | 0,46 | 0,64 | 0,89 |  | 0,27 | 0,59 | 0,78 | 0,96 |
|  |  |  |  |  |  |  |  |  |  |  |  |  |  |  |  |  |  |  |  |  |  |  |  |  |
| **Region of residence** |  |  |  |  |  |  |  |  |  |  |  |  |  |  |  |  |  |  |  |  |  |  |  |  |
| Mid-west | 0,19 | 0,41 | 0,60 | 0,90 |  | 0,44 | 0,67 | 0,79 | 0,95 |  | 0,12 | 0,32 | 0,54 | 0,86 |  | 0,19 | 0,35 | 0,46 | 0,78 |  | 0,21 | 0,45 | 0,65 | 0,93 |
| Northeast | 0,15 | 0,39 | 0,59 | 0,88 |  | 0,27 | 0,51 | 0,68 | 0,91 |  | 0,11 | 0,34 | 0,55 | 0,88 |  | 0,09 | 0,25 | 0,42 | 0,77 |  | 0,20 | 0,50 | 0,71 | 0,93 |
| Norht | 0,12 | 0,29 | 0,46 | 0,81 |  | 0,32 | 0,55 | 0,71 | 0,92 |  | 0,08 | 0,23 | 0,39 | 0,78 |  | 0,14 | 0,27 | 0,38 | 0,67 |  | 0,18 | 0,42 | 0,61 | 0,90 |
| Southeast | 0,16 | 0,40 | 0,61 | 0,91 |  | 0,41 | 0,63 | 0,77 | 0,95 |  | 0,08 | 0,27 | 0,49 | 0,87 |  | 0,14 | 0,37 | 0,55 | 0,84 |  | 0,20 | 0,46 | 0,68 | 0,94 |
| South | 0,21 | 0,49 | 0,69 | 0,93 |  | 0,30 | 0,62 | 0,80 | 0,95 |  | 0,14 | 0,40 | 0,61 | 0,91 |  | 0,15 | 0,34 | 0,54 | 0,87 |  | 0,25 | 0,54 | 0,74 | 0,95 |
|  |  |  |  |  |  |  |  |  |  |  |  |  |  |  |  |  |  |  |  |  |  |  |  |  |
| **Clinical stage** |  |  |  |  |  |  |  |  |  |  |  |  |  |  |  |  |  |  |  |  |  |  |  |  |
| I | 0,13 | 0,35 | 0,55 | 0,87 |  | 0,09 | 0,26 | 0,48 | 0,87 |  | 0,08 | 0,26 | 0,46 | 0,85 |  | 0,13 | 0,37 | 0,56 | 0,85 |  | 0,19 | 0,45 | 0,66 | 0,93 |
| II | 0,15 | 0,39 | 0,61 | 0,92 |  | 0,09 | 0,35 | 0,65 | 0,85 |  | 0,09 | 0,31 | 0,54 | 0,90 |  | 0,13 | 0,25 | 0,39 | 0,75 |  | 0,20 | 0,46 | 0,69 | 0,94 |
| III | 0,16 | 0,41 | 0,64 | 0,93 |  | 0,23 | 0,65 | 0,70 | 0,90 |  | 0,13 | 0,36 | 0,59 | 0,91 |  | 0,11 | 0,29 | 0,49 | 0,82 |  | 0,18 | 0,46 | 0,69 | 0,95 |
| IV | 0,24 | 0,52 | 0,72 | 0,94 |  | 0,19 | 0,50 | 0,74 | 0,90 |  | 0,16 | 0,42 | 0,64 | 0,92 |  | 0,17 | 0,37 | 0,53 | 0,83 |  | 0,26 | 0,56 | 0,76 | 0,95 |
|  |  |  |  |  |  |  |  |  |  |  |  |  |  |  |  |  |  |  |  |  |  |  |  |  |
| **Topography** |  |  |  |  |  |  |  |  |  |  |  |  |  |  |  |  |  |  |  |  |  |  |  |  |
| Central Nervous System | 0,35 | 0,60 | 0,75 | 0,94 |  |  |  |  |  |  |  |  |  |  |  |  |  |  |  |  |  |  |  |  |
| Cervix | 0,10 | 0,31 | 0,52 | 0,87 |  |  |  |  |  |  |  |  |  |  |  |  |  |  |  |  |  |  |  |  |
| Thyroid | 0,12 | 0,32 | 0,49 | 0,81 |  |  |  |  |  |  |  |  |  |  |  |  |  |  |  |  |  |  |  |  |
| Colorectal | 0,21 | 0,48 | 0,70 | 0,94 |  |  |  |  |  |  |  |  |  |  |  |  |  |  |  |  |  |  |  |  |
